# Outcomes of fertility preservation in women with endometriosis: comparison of progestin-primed ovarian stimulation versus antagonist protocols

**DOI:** 10.1186/s13048-020-00620-z

**Published:** 2020-02-13

**Authors:** Emmanuelle Mathieu d’Argent, Clément Ferrier, Chrysoula Zacharopoulou, Naouel Ahdad-Yata, Anne-Sophie Boudy, Adèle Cantalloube, Rachel Levy, Jean-Marie Antoine, Emile Daraï, Sofiane Bendifallah

**Affiliations:** 1 Department of Gynaecology and Obstetrics, Tenon University Hospital. Assistance Publique des Hôpitaux de Paris (AP-HP). Faculty of Medicine Pierre and Marie Curie. Sorbonne University, Paris, France; 2 Groupe de Recherche Clinique GRC6-UPMC : Centre Expert En Endométriose (C3E), Paris, France; 3 Department of Gynaecology and Obstetrics. Military hospital Bégin, Saint-Mandé, France; 4 Department of Histology, Embryology, cytogenetic, CECOS. Hôpital Jean Verdier. Assistance Publique des Hôpitaux de Paris (AP-HP), Bondy, France

**Keywords:** Cryopreservation, Endometriosis, Assisted-reproductive technology, Cost-effectiveness, Infertility

## Abstract

**Background:**

PPOS protocols, initially described for FP in women with cancer, have many advantages compared to antagonist protocols. PPOS protocols were not evaluated for women with endometriosis. The objective of the study was to describe fertility preservation outcomes in women with endometriosis and to compare an antagonist protocol with a Progestin-Primed Ovarian Stimulation (PPOS) protocol.

**Method:**

We conducted a prospective cohort study associated with a cost-effectiveness analysis in a tertiary-care university hospital. The measured outcomes included the numbers of retrieved and vitrified oocytes, and direct medical costs. In the whole population, unique and multiple linear regressions analysis were performed to search for a correlation between individual characteristics and the number of retrieved oocyte.

**Results:**

We included 108 women with endometriosis who had a single stimulation cycle performed with either an antagonist or a PPOS protocol. Overall, 8.1 ± 6.6 oocytes were retrieved and 6.4 ± 5.6 oocytes vitrified per patient. In the multiple regression model, age (*p* = 0.001), prior ovarian surgery (*p* = 0.035), and anti-Mullerian hormone level (*p* = 0.001) were associated with the number of retrieved oocytes. Fifty-four women were stimulated with an antagonist protocol, and 54 with a PPOS protocol. A mean of 7.9 ± 7.4 oocytes were retrieved in the antagonist group and 8.2 ± 5.6 in the PPOS group (*p* = 0.78). A mean of 6.4 ± 6.4 oocytes were vitrified in the antagonist group and 6.4 ± 4.7 in the PPOS group (*p* = 1). In the cost-effectiveness analysis, the PPOS protocol was strongly dominant over the antagonist protocol.

**Conclusion:**

Fertility preservation procedures are feasible and effective for patients affected by endometriosis. Antagonist and PPOS protocols were associated with similar results but the medico-economic analysis was in favor of PPOS protocols.

## Background

Endometriosis represents a major public health challenge. Although its incidence is still difficult to assess accurately, it is thought to affect 10% of women of reproductive age worldwide and up to 40 to 50% of infertile women. In France, an estimated 2.1 to 4.2 million women are affected by endometriosis which is responsible for 30 to 40% of cases of hypo- or infertility [1–4].

In France, fertility preservation (FP) is authorized and has been fully covered by the healthcare system since the 2004 Bioethics Laws (modified in 2011) especially for women with potentially fertility-associated disorders such as endometriosis [5, 6]. At the same time, with the worldwide trend of delaying childbearing in developed countries [7–9], the motivation for FP is growing.

Among the various techniques reported for FP (oocyte cryopreservation, freezing of embryos, and ovarian cortex cryopreservation) [10], oocyte cryopreservation has been reported to be the best option because of its low negative impact on ovarian reserve and low associated morbidity compared to other FP techniques [11].

In this setting, several ovarian stimulation protocols, originally developed for assisted reproduction technology (ART), have been used for FP especially for women with cancer. Recently, Kuang et al. described a progestin-primed ovarian stimulation (PPOS) protocol using exogenous progesterone to replace the GnRH agonist or antagonist in the follicular phase [12]. In their retrospective cohort study, compared with an antagonist short protocol the number of mature oocytes retrieved and the number of frozen embryos were not significantly different [13]. However, to the best of our knowledge, published data assessing the most effective stimulation protocol in the context of endometriosis are scarce.

Therefore, the aim of the current study was to analyze FP outcomes in women with endometriosis and to compare an antagonist protocol with a PPOS protocol in terms of FP outcome (retrieved and vitrified oocytes) and cost-effectiveness.

## Methods

### Study setting and patients

The data of all women diagnosed with endometriosis and referred for FP from July 2015 to October 2018 were extracted from the prospectively maintained database of Tenon University Hospital.

Inclusion criteria for FP were: i) age < 40 years, ii) endometriosis with ovarian cysts with and without deep endometriosis (DE), and iii) alteration of ovarian reserve (antral follicle count (AFC) < 10 and/or anti-Mullerian hormone (AMH) level < 2). Indications for FP were systematically validated by a multidisciplinary committee.

### Procedures

After having received relevant information and participated in a conference explaining the modalities of FP, each patient could choose whether to undergo a single cycle of the antagonist or PPOS protocol. Any patient on long-term oral progestin treatment who chose the antagonist protocol had to stop her progestin treatment and the protocol was started on the 1st or 2nd day of a natural cycle. For the PPOS protocol, patients could continue their long-term oral progestin treatment and start the protocol when they wished. Patients in the PPOS protocol without long-term oral progestin treatment started an oral treatment by desogestrel at the same time as ovarian stimulation, on the 1st day of a natural cycle. In both groups, gonadotropin (150/450 UI FSH or HMG) was injected daily according to body mass index (BMI), AMH and AFC. The dose of gonadotropins was monitored and adapted using transvaginal ultrasonography (assessing follicle number and size) and blood tests (luteinizing hormone (LH), estradiol (E2) and progesterone serum levels). In the antagonist protocol group, a GnRH antagonist (Ganirelix, orgalutran® MSD France) was injected daily from day 6 to the trigger day. When three dominant follicles reached 18 mm in diameter, the final stage of oocyte maturation was triggered with the use of 0.2 mg triptorelin (Decapeptyl; Ferring Pharmaceuticals) or Ovitrelle® 250 μg (Serono Europe limited lab). Transvaginal ultrasound-guided oocyte retrieval was performed 34–36 h after the trigger. All follicles with a diameter > 10 mm were retrieved. The aspirated oocytes were frozen by vitrification on the day of retrieval.

### Outcome measures

The primary outcome measure was the number of oocytes retrieved during the cycle. Secondary measures included the number vitrified oocytes, the rate of moderate/severe ovarian hyperstimulation, the rate of cycle cancellation due to insufficient ovarian response and the rate of LH surge incidence (LH level > 10 UI/l).

### Cost-effectiveness analysis

We considered all direct medical costs related to the FP procedure. The treatment doses, number of consultations, blood tests performed, and number of transvaginal ultrasonography exams were recorded for each patient applying the national regulated price (in 2018) for each element [14, 15]. We assumed non-medical costs (transport, daily allowances …) to be zero. As FP procedures are fully covered by the public health insurance in France and no extra services were charged, we also considered out-of-pocket expenditure to be zero. The temporal horizon was the duration of the cycle. Consequently, actualization processing was not necessary [16].

### Statistical analysis

Patient characteristics, biological and sonographic parameters during the cycle and the number of oocytes retrieved and vitrified in each protocol were compared using Student test and χ^2^ tests. Statistical uni- and multivariate analysis were performed. For the medico-economic analysis, the protocols were compared on the basis of their effectiveness and costs. We performed all analysis using Stata/IC 14.0 and Excel 2013 software.

### Ethical approval

Prospective recording of data was approved by the French Authority, the Advisory Committee on Information Processing in Healthcare Research (ID-RCB: 2018-A01141–54).

## Results

### Characteristics of the population

**Fig. 1:**
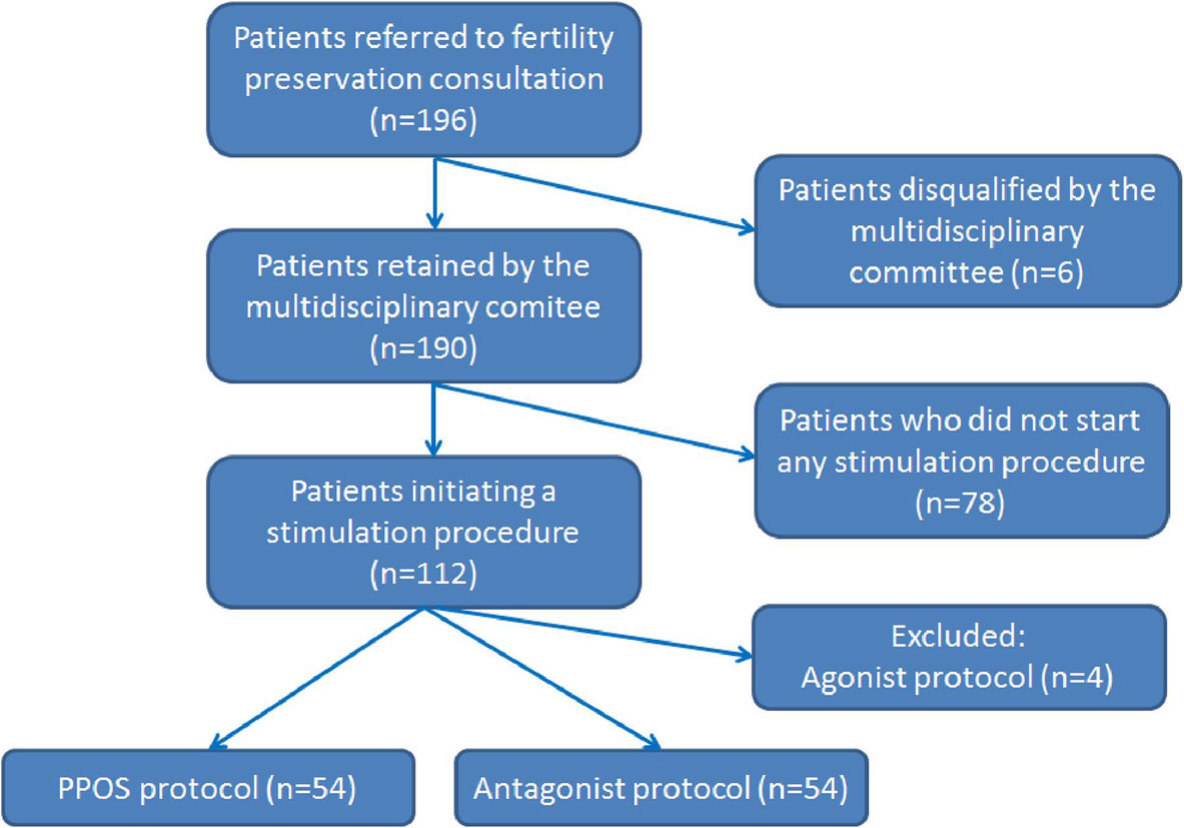
Flow-chart

A total of 196 women were referred for FP during the study period. Among them, 108 fulfilled the inclusion criteria and constituted the study population (Fig. 1: flow chart).

The mean age was 30.3 years, mean serum AMH level 2.0 ng/ml, and mean AFC 11.3. Nearly all of the women (92%) had DE including 42.7% with digestive involvement. Moreover, 70.4% of the women had a history of endometrioma. Before stimulation, 88 patients (82.2%) were on oral progestin treatment. For the PPOS group, the progestin was promegestone (*n* = 23), chlormadinone (*n* = 22), medrogestone (*n* = 3), acetate of nomegestrol (*n* = 3), or desogestrel (*n* = 3). Patient characteristics were statistically comparable between the two groups (Table 1).

**Table 1:** Characteristics of the study population

	Total (*n* = 108)	Antagonist protocol (*n* = 54)	PPOS protocol (*n* = 54)	*p*-value
Age (years) (mean ± SD)	30.3 ± 4.3	30.8 ± 4.6	29.7 ± 4.0	0.19^1^
BMI (kg/m^2^)(mean ± SD)	22.2 ± 4.1	21.9 ± 2.6	22.6 ± 5.1	0.35^1^
Smoking (%(n))	31.5% (34/108)	33%	29%	0.68^2^
Positive gravidity (%(n))	20.5% (22/108)	22%	19%	0.42^2^
Positive parity (%(n))	6.5% (7/108)	6%	7%	0.70^2^
Prior ovarian surgery (%(n))	27.8% (30/108)	31%	24%	0.39^2^
Prior surgery for endometrioma (%(n))	21.5% (23/107)	26%	17%	0.22^2^
Prior surgery for endometriosis (%(n))	20.8% (22/106)	17%	25%	0.34^2^
AMH (ng/ml) (mean ± SD)	2.1 ± 1,9	2.1 ± 2.4	2 ± 1.3	0.82^1^
AFC (n) (mean ± SD)	11.3 ± 6,8	11.1 ± 7.4	11.5 ± 6.2	0.75^1^
Endometrioma during the FP (%(n))	34.3% (36/105)	31%	38%	0.45^2^
DE (%(n))	92.1% (93/101)	92%	92%	0.93^2^
DE with digestive involvement (%(n))	42.7% (41/96)	35%	49%	0.16^2^
Adenomyosis (%(n))	25.5% (25/98)	25%	27%	0.82^2^
Oral progestin treatment before FP(%(n))	82.2% (88/107)	76%	89%	0.08^2^

*BMI* Body mass Index, *AMH* Anti-Mullerian Hormone, *AFC* Antral Follicular Count, *DE* Deep Endometriosis, *SD* standard deviation, *FP* Fertility Preservation; *P*-value for the comparison between the antagonist protocol and the PPOS protocol; 1: t-test; 2: Chi2 test

### Outcomes

#### Ovarian stimulation

No significant differences were observed between the PPOS and antagonist protocols in terms of total dose of gonadotrophin (*p* = 0.281), duration of treatment (*p* = 0.58), trigger method (*p* = 0.071), E2 and progestin levels on the trigger day (*p* = 0.678 and *p* = 0.44, respectively). Stimulation parameters and hormone levels are reported in Table 2.

**Table 2:** Stimulation parameter, hormone levels and number of mature follicles on the trigger day (or trigger day-1), retrieved and vitrified oocytes

	Whole population (*n* = 108)	Antagonist protocol (*n* = 54)	PPOS protocol (*n* = 54)	*p*-value
Total dose of gonadotropins (UI) (mean ± SD)	3691 ± 1247	3826 ± 1340	3556 ± 1247	0.281^1^
Duration of the gonadotropin treatment (days) (mean ± SD)	11.3 ± 2.1	11.4 ± 2.0	11.2 ± 2.1	0.58^1^
Trigger:				0.071^2^
-hcg	12%	19%	6%	
-decapeptyl GnRH analogue	88%	81%	94%	
Estradiol level (pg/ml) (mean ± SD)	1281 ± 1392	1342 ± 1297	1219 ± 1494	0.678^1^
LH level (UI/L) (mean ± SD)	0.40 ± 0.63	0.44 ± 0.08	0.24 ± 0.06	0.03^1^
Progestin level (ng/ml) (mean ± SD)	1.5 ± 1.4	1.4 ± 1.2	1.6 ± 1.5	0.44^1^
Number of mature follicles (mean ± SD)	8.5 ± 5.2	8.3 ± 5.2	8.6 ± 5.2	0.745^1^
Number of retrieved oocytes (mean ± SD)	8.1 ± 6.6	7.9 ± 7.4	8.2 ± 5.6	0.78^1^
Number of vitrified oocytes (mean ± SD)	6.4 ± 5.6	6.4 ± 6.4	6.4 ± 4.7	1^1^

*SD* standart deviation; *P*-value for the comparison between the antagonist protocol and the PPOS protocol; 1: t-test; 2: chi2 test

#### Fertility preservation results (Table 2)

Overall, the protocols resulted in 8.1 ± 6.6 retrieved oocytes and 6.4 ± 5.6 vitrified oocytes per patient. The mean ± standard deviation of oocyte retrieved in the antagonist group and the PPOS group were 7.9 ± 7.4 and 8.2 ± 5.6 oocytes, respectively (*p* = 0.78). The mean ± standard deviation of oocytes retrieved in the antagonist group and the PPOS group were 6.4 ± 6.4 and 6.4 ± 4.7 oocytes, respectively (*p* = 1).

#### Multivariate regression model

In the multiple regression model, age (*p* = 0.001), prior ovarian surgery (*p* = 0.035), and the AMH level (*p* = 0.001) were associated with the number of retrieved oocytes. The presence of DE (versus superficial endometriosis alone), the location of endometriosis, the presence of endometrioma during the stimulation and the size of endometriomas were not associated with the number of retrieved oocytes (*p* > 0.05) (Table 3).

**Table 3:** Simple and multiple linear regression on the number of retrieved oocytes

Variable	Simple linear regression Coefficient 95%CI	*p*-value	Multiple linear regression Coefficient 95%CI	*p*-value
Age (years)	−0.23 [− 0.35: −.011]	< 0.001	− 0.48 [− 0.76: − 0.20]	0.001
BMI (kg/m^2^)	0.35 [− 0.08: 0.15]	0.56		
Tobacco use	−0.01 [− 0.02: 0.01]	0.22		
Prior pregnancy	0.20 [− 2.3: 2.7]	0.88		
Prior surgery for DE	0.52 [− 2.6: 3.7]	0.74		
Prior ovarian surgery	− 2.1 [− 4.9: 0.66]	0.13	−2.74 [− 5.28: − 0.20]	0.035
DE (vs. Superficial endometriosis only)	0.1 [− 3.9: 4.1]	0.96		
Presence of endometrioma	0.56 [− 2.1: 3.3]	0.68		
Presence of adenomyosis	0.44 [− 2.5: 3.4]	0.77		
AMH (ng/ml)	1.8 [1.2: 2.4]	< 0.001	1.29 [0.53: 2.05]	0.001
AFC (n)	0.38 [0.19: 0.57]	< 0.001	0.13 [− 0.09: 0.34]	0.248

*BMI* Body Mass Index, *DE* Deep Endometriosis, *AMH* Anti-Müllerian Hormone, *AFC* Antral Follicular Count; Tested variables in the simple linear regression model included: body mass index, tobacco consumption, gravidity, parity, history of surgery for DE, presence of DE (vs. superficial endometriosis only), presence of adenomyosis, presence of endometrioma, size of the biggest endometrioma, cumulative size of the endometriomas, long-term oral progestin treatment, PPOS protocol (vs. antagonist protocol)

#### Medico-economic analysis

No differences were observed between the protocols in terms of duration of the stimulation, the total dose of gonadotropin, the number of consultations (*n* = 2), the number of blood tests (*n* = 2) and the number of ultrasonography exams (*n* = 2) during the stimulation period. No complications occurred either during the stimulation or oocyte retrieval procedures. Consequently, the differences in costs between the two groups were for the GnRH antagonist for the antagonist group and the oral progestin treatment for the PPOS group.

For the antagonist group, the mean duration of GnRH antagonist treatment was 6.4 days (cost per day: €34). A total of 341 days of treatment was required amounting to a total cost of €11,600 (i.e. €215 per patient).

For the PPOS group, the mean duration of progestin treatment was 13.2 days (cost per day: €0.31). A total of 713 days of treatment was required amounting to a total cost of €319 (i.e. €6 per patient).

Thus, the incremental cost of the antagonist protocol was €11,281 (i.e. €209 per patient). Both protocols were equivalent in terms of PF results. Consequently, the PPOS protocol was strongly dominant over the antagonist protocol.

## Discussion

To our knowledge, this is the largest observational cohort study analyzing FP results for women with endometriosis, and the first to compare PPOS and antagonist protocols in this setting. We demonstrate that both protocols result in a similar number of retrieved oocytes and vitrified mature oocytes. In addition, in the whole population, age, prior ovarian surgery, and the AMH level were strong prognostic factors of PF outcome. The incremental cost of the PPOS protocol was €11,281 (€209 per patient) for the antagonist protocol making the PPOS protocol strongly dominant over the antagonist protocol.

The role of FP for patients with endometriosis is a major issue. The association between endometriosis and infertility is well recognized [4, 17], and several mechanisms have been implicated. The progressive loss of the follicle reservoir either spontaneously or after ovarian surgery is frequently observed [18, 19] and is often responsible for impaired fecundity, poor results in ART and an increased risk of premature ovarian failure. In this specific setting, we report the largest cohort (*n* = 108) to assess FP outcome for women with endometriosis. In 2018, Raad et al. published a retrospective cohort study of 62 women with endometriosis [20]. Forty-nine patients completed at least one cycle with similar stimulation parameters as in our study (mean stimulation duration: 11.1 (±2.7) days; mean dose of gonadotropin used: 3634 (±1656) IU). In their series, the mean number of retrieved and vitrified oocytes were 9.5 (±6.1) and 7.2 (±4.9), respectively. In contrast to Raad et al.’s series, our patients were younger (30.3 vs. 33.9 years), more often affected by DE (92% vs. 45%) and had lower AMH levels (2.1 vs. 2.3 ng/ml) and AFC (11.3 vs 13.0 follicles), potentially explaining the lower number of retrieved and vitrified oocytes in the current study. In the multivariate analysis, we also demonstrated that age, prior ovarian surgery and AMH levels were independent predictive factors of FP outcome. We thus suggest that FP should also be considered in patients at early stages of endometriosis when a reduced ovarian reserve is identified.

Several reports have studied the use of PPOS protocols for in vitro fertilization (IVF) procedures for women with endometriosis-associated infertility and found them to be more effective than other stimulation protocols [12, 21–26]. Indeed, a PPOS protocol has several advantages. It requires fewer injections than a conventional protocol, and may thus be considered more “user-friendly” for the patients. Moreover, patients with symptomatic endometriosis are usually under progestin treatment that can be continued for the initiation of a PPOS protocol. Finally, it should avoid the occurrence of painful menstruations before the ovarian stimulation, improving the tolerance of the procedure. PPOS protocols are thus easier to plan for both the physician and the patient. The PPOS protocol is also significantly cheaper, saving €209 per patient, and consequently strongly dominant over the antagonist protocol. We observed that the mean number of vitrified oocytes was 6.4 oocytes. Considering that the overall survival rate of vitrified oocytes has previously been evaluated at 85.2% (95% CI 83.2–87.2) [27], and that at least 8–10 metaphase II oocytes are required to achieve a pregnancy [27], most patients will require at least two cycles of ovarian stimulation for an effective FP, reinforcing the efficiency of the PPOS protocol.

The issue of FP for women with endometriosis is attracting interest worldwide. In 2018, the National French College of Obstetrics and Gynaecology (CNGOF) included FP in the guidelines for endometriosis management. These guidelines state that FP should be proposed to: (1) patients with endometrioma, especially in the case of bilateral endometriomas or a history of ovarian surgery and, (2) patients with isolated DE [28]. However, these recommendations are based on limited existing literature data and the indications deserve to be clarified by continuous data collection together with growing experience with FP in the context of endometriosis, taking both the economic burden and success rates into consideration.

In our cohort, a large part (41%) of the patients referred for a FP consultation and retained by the multidisciplinary committee had not undergone an FP procedure at the time of the study. This is somewhat surprising given that FP procedures are fully covered by the public health insurance in France. Nevertheless, we do not know how many of these patients (1) refused to undergo an FP procedure or (2) had planned a procedure which had not been performed at the time of the study. The causes for potential refusal should be explored in future studies to better select patients (and hence to decrease the cost of exams required for planning an FP procedure), and to improve patient information about the objectives and interests of FP in endometriosis.

Our study has several limitations. First, it was not a randomized-controlled trial and the choice of a protocol was based on patients’ preferences. The number of patients in each group was the same because of chance. Even though the two groups were comparable on observable parameters, we cannot exclude that unobservable parameters may differ and bias the results. Second, during the initial evaluation most of the patients were on hormonal treatment (80% were taking oral progestin). The effect of hormonal treatment on AMH level remains a matter of debate [29], but few studies suggest that progestin drugs tend to decrease AMH levels [30]. Third, we chose the number of retrieved and vitrified oocytes as outcome measures. No data about the oocyte quality or pregnancy outcomes after fertilization were available due to the short follow-up. Four, we assumed direct non-medical and productivity costs to be zero in our medico-economic analysis. Indeed, these data were unavailable in the database and no estimation could be extracted from the literature. However, in our experience, no physician prescribes medical transportation or sick leave for FP.

## Conclusion

Our results highlight that FP procedures are feasible and effective for patients with endometriosis, even if several cycles will be required for most patients. Antagonist and PPOS protocols are associated with similar outcome in terms of number of retrieved and vitrified oocytes with a medico-economic analysis in favor of PPOS protocols. We suggest that data should be collected on a continuous basis to further clarify indications for FP in the context of endometriosis, taking both the economic burden and success rates into consideration.

## Data Availability

The datasets used and/or analysed during the current study are available from the corresponding author on reasonable request.

## References

[1] Giudice LC, Kao LC (2004). Endometriosis. Lancet Lond Engl.

[2] Eskenazi B, Warner ML (1997). Epidemiology of endometriosis. Obstet Gynecol Clin North Am juin.

[3] Dunselman GA, Vermeulen N, Becker C, Calhaz-Jorge C, D’Hooghe T, De Bie B (2014). ESHRE guideline: management of women with endometriosis. Hum Reprod Oxf Engl.

[4] Meuleman C, Vandenabeele B, Fieuws S, Spiessens C, Timmerman D, D’Hooghe T (2009). High prevalence of endometriosis in infertile women with normal ovulation and normospermic partners. Fertil Steril juill.

[5] Loi n° 2004–800 du 6 août 2004 relative à la bioéthique. Available at: https://www.legifrance.gouv.fr/affichTexte.do?cidTexte=JORFTEXT000000441469. Accessed 15 Apr 2019.

[6] LOI n° 2011–814 du 7 juillet 2011 relative à la bioéthique. Available at: https://www.legifrance.gouv.fr/affichTexte.do?cidTexte=JORFTEXT000024323102. Accessed 15 Apr 2019.

[7] Centers for Disease Control and Prevention (CDC), 2011. Reproductive health. Infertility FAQs. Available at: <http://www.cdc.gov/reproductivehealth/Infertility/index.htm> (Accessed 15 Apr 2019).

[8] SOGC (2012). Society of obstetrician and gynaecologists of Canada: opinion committee, delayed childbearing. J Obstet Gynaecol Can.

[9] Volant. Un premier enfant à 28,5 ans en 2015 : 4,5 ans plus tard qu’en 1974. Available at: https://www.insee.fr/fr/statistiques/2668280. Accessed 15 Apr 2019.

[10] Donnez J, Dolmans M-M (2017). Fertility Preservation in Women. N Engl J Med.

[11] Cobo A, Diaz C (2011). Clinical application of oocyte vitrification: a systematic review and meta-analysis of randomized controlled trials. Fertil Steril.

[12] Kuang Y, Chen Q, Fu Y, Wang Y, Hong Q, Lyu Q (2015). Medroxyprogesterone acetate is an effective oral alternative for preventing premature luteinizing hormone surges in women undergoing controlled ovarian hyperstimulation for in vitro fertilization. Fertil Steril.

[13] Wang N, Wang Y, Chen Q, Dong J, Tian H, Fu Y (2016). Luteal-phase ovarian stimulation vs conventional ovarian stimulation in patients with normal ovarian reserve treated for IVF: a large retrospective cohort study. Clin Endocrinol (Oxf).

[14] Base de donnée publique des médicaments. Available at: https://solidarites-sante.gouv.fr/soins-et-maladies/medicaments. Accessed 15 Nov 2018.

[15] CCAM. Classification Commune des Actes Médicaux. Available at: https://www.ameli.fr/accueil-de-la-ccam/index.php. Accessed 15 Nov 2018.

[16] Department of economics and public health assessment. Choices in methods for economic evaluation. 2012. https://www.has-sante.fr/portail/jcms/r_1499251/fr/choix-methodologiques-pour-l-evaluation-economique-a-la-has. Accessed 13 Mar 2019.

[17] Prescott J, Farland LV, Tobias DK, Gaskins AJ, Spiegelman D, Chavarro JE (2016). A prospective cohort study of endometriosis and subsequent risk of infertility. Hum Reprod Oxf Engl.

[18] Seyhan A, Ata B, Uncu G (2015). The impact of endometriosis and its treatment on ovarian reserve. Semin Reprod Med.

[19] Goodman LR, Goldberg JM, Flyckt RL, Gupta M, Harwalker J, Falcone T (2016). Effect of surgery on ovarian reserve in women with endometriomas, endometriosis and controls. Am J Obstet Gynecol.

[20] Raad J, Sonigo C, Tran C, Sifer C, Durnerin IC, Grynberg M (2018). Oocyte vitrification for preserving fertility in patients with endometriosis: first observational cohort study … and many unresolved questions. Letter to the Editor. Eur J Obstet Gynecol Reprod Biol.

[21] Wang Y, Chen Q, Wang N, Chen H, Lyu Q, Kuang Y (2016). Controlled Ovarian Stimulation Using Medroxyprogesterone Acetate and hMG in Patients With Polycystic Ovary Syndrome Treated for IVF: A Double-Blind Randomized Crossover Clinical Trial. Medicine (Baltimore).

[22] Zhu X, Zhang X, Fu Y (2015). Utrogestan as an effective oral alternative for preventing premature luteinizing hormone surges in women undergoing controlled ovarian hyperstimulation for in vitro fertilization. Medicine (Baltimore).

[23] Zhu X, Ye H, Fu Y (2017). Comparison of neonatal outcomes following progesterone use during ovarian stimulation with frozen-thawed embryo transfer. Sci Rep.

[24] Iwami N, Kawamata M, Ozawa N, Yamamoto T, Watanabe E, Moriwaka O (2018). New trial of progestin-primed ovarian stimulation using dydrogesterone versus a typical GnRH antagonist regimen in assisted reproductive technology. Arch Gynecol Obstet.

[25] Huang C-Y, Chen G-Y, Shieh M-L, Li H-Y (2018). An extremely patient-friendly and efficient stimulation protocol for assisted reproductive technology in normal and high responders. Reprod Biol Endocrinol RBE.

[26] Yu S, Long H, Chang HY-N, Liu Y, Gao H, Zhu J (2018). New application of dydrogesterone as a part of a progestin-primed ovarian stimulation protocol for IVF: a randomized controlled trial including 516 first IVF/ICSI cycles. Hum Reprod Oxf Engl.

[27] Cobo A, García-Velasco JA, Coello A, Domingo J, Pellicer A, Remohí J (2016). Oocyte vitrification as an efficient option for elective fertility preservation. Fertil Steril.

[28] Decanter C, d’Argent EM, Boujenah J, Poncelet C, Chauffour C, Collinet P (2018). Endometriosis and fertility preservation: CNGOF-HAS Endometriosis Guidelines. Gynecol Obstet Fertil Senol.

[29] D’Arpe S, Di Feliciantonio M, Candelieri M, Franceschetti S, Piccioni MG, Bastianelli C (2016). Ovarian function during hormonal contraception assessed by endocrine and sonographic markers: a systematic review. Reprod Biomed Online.

[30] Birch Petersen K, Hvidman HW, Forman JL, Pinborg A, Larsen EC, Macklon KT (2015). Ovarian reserve assessment in users of oral contraception seeking fertility advice on their reproductive lifespan. Hum Reprod Oxf Engl.

